# Croup

**Published:** 1852-09

**Authors:** 


*Croup—Compound Tincture of Chloroform as a Remedy.* —(*Boston Soc. for Med. Improvement.*)—Dr. Strong was consulted, late on Sunday evening, in reference to a case of alleged croup. The parents had been familiar with the disease. After inquiring respecting the symptoms, it was thought best to send directions for the treatment, with the understanding that it should be tried, and if without any relief, Dr. S. was to be summoned in the night—otherwise, to visit next morning. The child is about two years old. The prescription for the night was hydrargyri subsulphatis ℈ss.; pulvis ipecacuanhæ ℈j.; pulvis ipecac. et opii, gr. vj.; misce et div. in chart. no. vj.; one powder to be given every fifteen minutes until free vomiting was induced; the feet to be first placed in warm water; and in case of no relief, or if there should be a temporary relief, and then a return of symptoms, the same to be repeated after an interval of two hours. Dr. S. saw the child the next forenoon, and learned that the emetic had been taken, with some temporary relief; the child, at his visit, had quick, laborious breathing, with a croupy cough, aggravated from time to time, and the peculiar dry, sonorous character of the respiration incident to the worst forms of croup; the countenance at times livid. The throat, carefully examined by sunlight, showed white lymph upon both tonsils and over the fauces. Having treated other cases like this without avail, Dr. S. was strongly impressed with the idea that the child would die, and so expressed himself. A cathartic was prescribed, the bowels being confined; and compound tincture of chloroform was freely applied, by cloths saturated with it, to the neck, and the fauces and tonsils were wetted with it by means of a linen "swab." The cloths were re-moistened and applied as soon as they became dry; and this was persevered in through the day, the applications and swabbing being attended to perhaps as often as once an hour. At a visit made that evening, the same treatment was directed for the ensuing night, with the addition of a grain of Dover's powder, which was to be repeated every two to four hours, as the symptoms were, or were not urgent.

Next morning the child was better, there had been no attacks of severe dyspnœa; and there were intervals when the breathing was nearly natural. The same treatment was still continued; the application to the throat to be made at longer intervals; in the evening of this day all the croupy symptoms were gone, and the child recovered rapidly. Auscultation detected evidences of bronchitis.

The compound tincture of chloroform has been used by Dr. S. in incipient inflammations—as in bubo, in inflamed glands of the neck, paronychia, and deep-seated inflammation of the hand; also, in inflamed and ulcerated gums; and, so far, with most marked advantage.—*Amer. Jour. Med. Sci.*

